# In Silico Drug Repurposing Framework Predicts Repaglinide, Agomelatine and Protokylol as TRPV1 Modulators with Analgesic Activity

**DOI:** 10.3390/pharmaceutics14122563

**Published:** 2022-11-22

**Authors:** Corina Andrei, Dragos Paul Mihai, Anca Zanfirescu, George Mihai Nitulescu, Simona Negres

**Affiliations:** Faculty of Pharmacy, “Carol Davila” University of Medicine and Pharmacy, Traian Vuia 6, 020956 Bucharest, Romania

**Keywords:** molecular docking, machine learning, pain, drug discovery, TRPV1 agonist, TRPV1 antagonist

## Abstract

Pain is one of the most common symptoms experienced by patients. The use of current analgesics is limited by low efficacy and important side effects. Transient receptor potential vanilloid-1 (TRPV1) is a non-selective cation channel, activated by capsaicin, heat, low pH or pro-inflammatory agents. Since TRPV1 is a potential target for the development of novel analgesics due to its distribution and function, we aimed to develop an in silico drug repositioning framework to predict potential TRPV1 ligands among approved drugs as candidates for treating various types of pain. Structures of known TRPV1 agonists and antagonists were retrieved from ChEMBL databases and three datasets were established: agonists, antagonists and inactive molecules (pIC50 or pEC50 < 5 M). Structures of candidates for repurposing were retrieved from the DrugBank database. The curated active/inactive datasets were used to build and validate ligand-based predictive models using Bemis–Murcko structural scaffolds, plain ring systems, flexophore similarities and molecular descriptors. Further, molecular docking studies were performed on both active and inactive conformations of the TRPV1 channel to predict the binding affinities of repurposing candidates. Variables obtained from calculated scaffold-based activity scores, molecular descriptors criteria and molecular docking were used to build a multi-class neural network as an integrated machine learning algorithm to predict TRPV1 antagonists and agonists. The proposed predictive model had a higher accuracy for classifying TRPV1 agonists than antagonists, the ROC AUC values being 0.980 for predicting agonists, 0.972 for antagonists and 0.952 for inactive molecules. After screening the approved drugs with the validated algorithm, repaglinide (antidiabetic) and agomelatine (antidepressant) emerged as potential TRPV1 antagonists, and protokylol (bronchodilator) as an agonist. Further studies are required to confirm the predicted activity on TRPV1 and to assess the candidates’ efficacy in alleviating pain.

## 1. Introduction

Chronic pain is correlated with disability and significant impairment of the patient’s quality of life. Furthermore, most of the patients need medical assistance to treat pain [1,2]. Depending on the etiology and type of the pain, current therapeutic solutions include drugs such as opioids, nonsteroidal anti-inflammatory, analgesic-antipyretics, antidepressants and anticonvulsant drugs. However, their use is limited by severe side effects and/or reduced efficacy [3,4,5]. Current research focuses on the discovery of new drugs with analgesic effect, with superior efficacy and safety profile [2].

In recent years, multiple studies have revealed the capsaicin receptor, the transient receptor potential vanilloid 1 (TRPV1), as a therapeutic target for the discovery of new analgesics [3,6,7,8,9,10].

The non-selective cation channel TRPV1 is highly expressed in peripheral unmyelinated C fibers and thinly myelinated A-delta fibers, and has lower levels of expression in upstream central components of the pain circuitry [11,12]. It is activated by low pH (<6.5), temperature > 42 °C and multiple exogenous (such as capsaicin, resiniferatoxin (RTX), gingerol and zingerone) and endogenous ligands (such as anandamide). The activation of this receptor was correlated with the appearance of inflammation and pain [13]. Capsazepine (CPZ), ruthenium red and iodo-resiniferatoxin are well-known TRPV1 receptor antagonists [14].

The role of the TRPV1 channel in pain modulation has been supported by numerous pharmacological and genetic studies [3]. Its activation enhances various cascades of intracellular signaling, involved in the response to algogenic and inflammatory agents [11]. In animal models using TRPV1 knockout mice, a significant decrease in thermal hyperalgesia associated with inflammatory pain has been observed [15,16]. Capsaicin administered in an increased dose (e.g., 6 nmol/rat administered intra-periaqueductal grey [17]) initially causes a sensation of irritation followed by loss of sensitivity to mechanical, chemical and thermal painful stimuli [18]. Thus, pharmaceutical preparations with agonists for different routes of administration are evaluated for the treatment of chronic pain [19,20]. 

**Figure 1:**
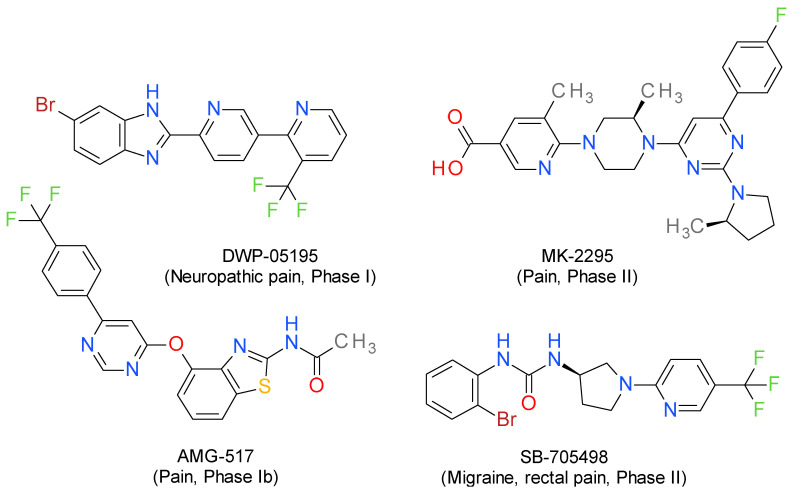
Representative structures of TRPV1 antagonists that have undergone clinical trials for pain management [8].

Owing to the specific side effects of TRPV1 agonists following oral administration (e.g., digestive irritation and pain), current research has focused on the discovery of new selective low molecular weight antagonists [3,21]. Antagonists (e.g., BCTC, AMG9810, A425619, and SB-705498) [11] and powerful agonists (such as capsaicin or RTX), that cause desensitization of TRPV1 [3,19], have been shown to attenuate thermal and mechanical hyperalgesia as well as tactile allodynia in various preclinical models—inflammatory [22,23,24,25], neuropathic [23,26], postoperative, cancer-related [27,28] and osteoarthritic pain [29,30]. Although some of the TRPV1 antagonists (Figure 1) were assessed in clinical phase I and phase II trials for the treatment of inflammatory, neuropathic and visceral pain, their severe hyperthermal side effects led to their withdrawal [31].

The discovery of new TRPV1 antagonists with fewer side effects is necessary for proper pain management. The structural details of the TRPV1 channel are essential for identifying such compounds. According to mutagenic studies, amino acid residues Tyr511 and Ser512 located in the loop between transmembrane domains (TM) 2 and 3, and Thr550 in the loop between TM4 and 5 are involved in mediating the effect of capsaicin on the TRPV1 channel [32,33]. However, Cao et al. analyzed the structure of resiniferatoxin- or capsaicin-bound TRPV1 and observed that these agonists bind to TM3-4, but also act on TM4-5 and 6 [34]. This is supported by different chimeric strategies that have revealed that TM3 and 4 are important for binding agonists (capsaicin and RTX) and antagonists (CPZ) [35]. 

Although the approval of analgesic drugs acting on the TRPV1 channel has been limited for the aforementioned reasons, information on the key elements of the receptor structure that contribute to its interactions is essential for the discovery of new therapeutic agents with analgesic potential. Drug repurposing studies are based on the discovery of new uses for approved or experimental drugs, and advantages such as reduced costs and time and low risk of failure support the use of this method [36]. A drug repurposing virtual screening framework was implemented in our study to identify, among approved drugs, new potential ligands that may interact with the TRPV1 receptor, considering that for these substances the pharmaco-toxicological profiles are well known.

Thus, the aim of our study was the use of in silico methods (ligand-based methods such as graph mining, classification models and structure-based methods based on molecular docking experiments) to identify potential TRPV1 antagonists and agonists/desensitizers with potential analgesic effects for the treatment of chronic pain. 

## 2. Materials and Methods

**Figure 2:**
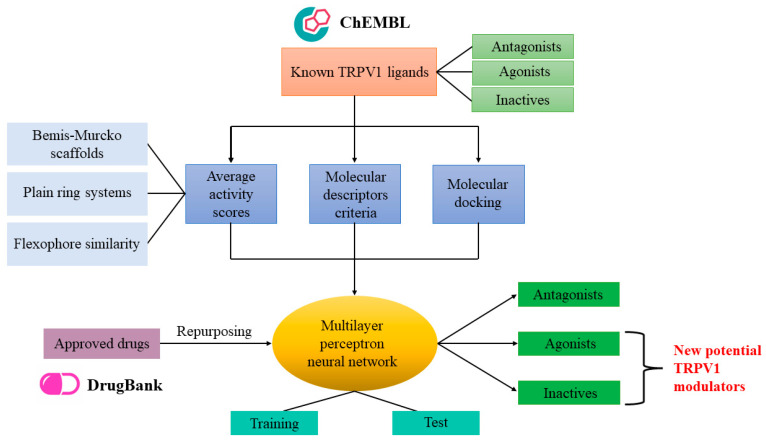
Schematic illustration of the drug repurposing framework.

A virtual screening framework was implemented with the scope of discovering potentially novel TRPV1 antagonists and agonists/desensitizers, using both ligand-based and structure-based in silico approaches. The proposed multi-step methodology is presented summarily in Figure 2. The implemented framework focused on building a machine learning algorithm (artificial neural network) based on structural scaffolds, flexophores, molecular descriptors and predicted binding affinities. The predictive model was used to find promising candidates as potential analgesic agents and is discussed in more detail in the following sections.

### 2.1. Datasets Curation

Chemical structures of known human TRPV1 antagonists and agonists with their corresponding activity values expressed as half maximal inhibitory concentration (IC_50_, nM) or half maximal effective concentration (EC_50_, nM) were downloaded from the ChEMBL database [37]. Datasets with activity values expressed as inhibitory constant (K_i_, nM), potency (nM), activity (%) or inhibition (%) were also retrieved, to extract molecules that were further included in the inactive dataset (molecules declared as inactive in the database). Three datasets were built: active antagonists (ANT), active agonists (AG) and inactive molecules (IN). 

OSIRIS DataWarrior v5.0.0 software [38] was used to further curate the datasets. Mean IC_50_ or EC_50_ values were calculated for compounds tested in multiple activity assays, duplicate structures being merged into a single entry, and negative logarithmic values of IC_50_ (pIC_50_, M) and EC_50_ (pEC_50_, M) were calculated for all compounds, where applicable. The chemical structures with pIC_50_/pEC_50_ values lower than 5 M were saved separately as inactive ligands and were merged with the structures tagged as “inactive” in the Ki, potency, activity or inhibition datasets. Notably, some compounds were found within both the ANT and AG datasets, since agonists can act as desensitizers that inhibit the channel activity at higher concentrations, and have activity values expressed as both EC_50_ (for activating TRPV1) and IC_50_ (for desensitizing TRPV1), hence not being “true” antagonists [39]. These compounds were removed from the ANT dataset and were retained as agonists. A diverse decoy dataset was also created for the validation of the molecular docking protocol. The decoys were extracted from ChEMBL and were chosen based on common molecular descriptors widely used for describing drug-likeness, such as the molecular weight (MW), the logarithm of octanol/water partition coefficient (logP), the number of hydrogen bond donors (HBD) and the number of hydrogen bond acceptors (HBA). All these values had to be within the ranges of the active molecules. Three-dimensional coordinates were generated for all retained structures using OpenBabel v2.4.1 [40].

Compounds that were used for the drug repurposing screening framework were downloaded from the DrugBank v5.1.9 database [41] with their respective desalted 3D coordinates. The acquired database consisted only of approved drugs for human use and included neither organometallic nor biologic drugs.

Molecular descriptors (1D and 2D) were calculated with PaDEL-Descriptor v2.21 [42] and were integrated into both datasets for future analyses. Constant descriptors were removed from the datasets.

### 2.2. Ligand-Based Models

#### 2.2.1. Activity Scores

Activity scores were calculated for agonists, antagonists, inactives and decoys based on their activity values. These values were equal with pIC_50_/pEC_50_ for compounds in the AG and ANT sets, respectively, and were considered 0 for inactives and decoys. Further, structural scaffolds were taken into account by generating Bemis–Murcko skeletons and plain ring systems with DataWarrior. Bemis–Murcko (BM) skeletons are molecular frameworks that result from the removal of atom types, bond types and side chains, and have proven to be useful in various in silico screening studies [43,44]. Plain ring systems (PR) are rings with removed substitution patterns, linkers and side chains [45,46].

Flexophore descriptors were generated for all datasets using DataWarrior. A flexophore is a 3D versatile pharmacophore descriptor calculated based on molecular flexibility, which is represented using a complete graph. The function compares vertices and edges between maximum common substructures of two descriptor graphs [47]. Within each set, compounds were clustered based on flexophores with an 80% similarity threshold.

Activity scores were generated for each specific BM skeleton, PR and flexophore cluster by calculating the arithmetic means of activity values. The average activity score (*Score*) of each individual compound was calculated as the average of BM scores (*BMS*), PR scores (*PRS*) and flexophore similarity clusters scores (*SCS*) (Equations (1)–(4)).

(1) $$SCS=\frac{1}{n}\sum_{1}^{n}pX$$

(2) $$BMS=\frac{1}{n}\sum_{1}^{n}pX,\;\mathrm{if}\;{\mathrm{BM}}_{x}\;\mathrm{is}\;\mathrm{present},\;\mathrm{else}\;BMS=0$$

(3) $$PRS=\frac{1}{i}\left(\frac{1}{n}\sum_{1}^{n}pX+\frac{1}{m}\sum_{1}^{m}pX+\cdots +\frac{1}{z}\sum_{1}^{z}pX\right),\;\mathrm{if}\;{\mathrm{PR}}_{x}\;\mathrm{are}\;\mathrm{present},\;\mathrm{else}\;PRS=0$$

(4) $$Score=\frac{BMS+PRS+FRS}{3}$$

where *i*—number of plain rings,

*pX*—pIC_50_ or pIC_50_; 0 for inactives.

#### 2.2.2. Binary Classification Model

Binary classification models were built based on setting cutoff values for several descriptors, similar to our previous work [48]. An independent sample *t*-test was applied in order to identify molecular descriptors that were statistically different between active and inactive molecules. Descriptors with areas under the receiver operating characteristics (ROC) curve >0.8 were further processed by building the correlation matrix and were referred to as variables. Variables that were highly intercorrelated (R > 0.75) were excluded. Cutoff values of the classifiers were chosen using ROC curves and by identifying the coordinates with a good balance between sensitivity and specificity. Classification performance parameters were calculated (sensitivity, specificity, accuracy, ROC AUC and F1 score) for model evaluation. The classification model was applied thereafter to the DrugBank dataset.

### 2.3. Structure-Based Approaches

Molecular docking experiments were carried out to estimate the predicted binding affinity of screened molecules to the TRPV1 channel. The crystal structures of human TRPV1 in complex with either RTX or CPZ were retrieved from the RCSB Protein Data Bank (PDB codes: 5IS0 [49] and 7MZC [50]) and were further optimized using YASARA Structure [51], by removing solvent molecules, correcting structural errors, adding missing residues and polar hydrogens at physiological pH (7.4), and by optimizing the hydrogen-bonding networks. The retrieved protein–ligand complexes were thereafter minimized using the NOVA2 forcefield.

The docking protocols were validated by extracting the co-crystallized ligands and redocking them into the binding sites. Thereafter, the predicted conformations of the ligands were superposed on the experimentally determined structure and the root-mean-square deviation (RMSD) values were calculated. Ligands used for validation also served as positive controls in terms of the correct binding pose.

Three-dimensional structures of TRPV1 agonists and antagonists, inactive molecules, decoys and DrugBank compounds were prepared for docking using energy minimization with the MMFF94s+ force field and protonation at physiological pH. Docking runs were executed using the AutoDock Vina v1.1.2 algorithm [52], and the grid box was selected based on the coordinates of the co-crystallized ligands. For the validation of the docking experiment in terms of prioritizing active ligands over inactive molecules and for balancing the datasets, a selection of antagonists and inactive compounds was performed based on structural similarity and clustering, as suggested by expert opinions on molecular docking-based virtual screening protocols [53], while all agonists were retained. Therefore, ligands were clustered using a 0.75 structural similarity threshold and only the most potent compound from each cluster was retrieved. Validation of the docking score accuracy was performed through ROC curve analysis.

Docking scores (binding energies, ΔG, kcal/mol) and ligand efficiencies (LE, ΔG/no. of heavy atoms) corresponding to the first conformation generated for each ligand were retrieved for the screened compounds (TRPV1 agonists and antagonists, inactive molecules, decoys and repositioning candidates). Graphical depictions of ligand poses and molecular interactions were generated using BIOVIA Discovery Studio Visualizer (BIOVIA, Discovery Studio Visualizer, Version 17.2.0, Dassault Systèmes, 2016, San Diego, CA, USA). 

### 2.4. Integrated Repurposing Model

A global predictive model was applied for all retrieved DrugBank molecules by integrating average activity scores, satisfied descriptor criteria and docking scores. The repurposing model was a multi-class classification algorithm based on a multilayer perceptron neural network (MLP NN) and was trained to discriminate not only between active and inactive molecules but also between TRPV1 agonists and antagonists. The ChEMBL datasets were randomly split into training (70%) and test (30%) subsets for model training and validation. This dataset included all the agonists, but only the antagonists and inactive molecules selected for the molecular docking study. Therefore, the ratio between antagonists, agonists and inactive ligands was approximately 1:1:2, which is relatively balanced. The network input layer consisted of the normalized values of the 6 dependent variables. The most optimal network architecture was established by varying the number of hidden layers and neurons in each layer. Moreover, hyperparameters such as initial learning rate, momentum and the maximum number of epochs were also varied until the most optimal model was generated. A gradient descent algorithm was used for optimization. Since we were dealing with a multi-class classification problem, hyperbolic tangent (tanh) activation functions were used for the hidden layers, and the softmax activation function was used for the output layer.

### 2.5. Statistical Analysis, Machine Learning and Performance Metrics

ROC curve analysis, independent *t*-tests, correlation analysis and MLP NN generation were performed using IBM SPSS Statistics v20 (Armonk, New York, NY, USA). Performance metrics of binary classification and logistic regression models were calculated using the following equations:

(5) $$ACC=\frac{TP+TN}{TP+TN+FP+FN}$$

(6) $$TPR=\frac{TP}{TP+FN}$$

(7) $$TNR=\frac{TN}{TN+FP\;}\;$$

(8) $$F1=\frac{2TP}{2TP+FP+FN},$$

where *ACC* = accuracy;

*TPR*—true positive rate (recall or sensitivity);

*TNR*—true negative rate (selectivity or specificity);

*F*1—F-score (harmonic mean of precision and recall);

*TP*—true positives;

*TN*—true negatives;

*FP*—false positives;

*FN*—false negatives.

## 3. Results

### 3.1. Dataset Preparation

Virtual chemical libraries were constructed in order to implement the in silico drug repurposing campaign. After initial curation, the datasets contained the chemical structures and activity values of 2377 TRPV1 antagonists (ANT set), 194 agonists (AG set), and 996 experimentally determined inactive molecules (IN set). In order to establish a set of decoy molecules with matching properties with the active molecules, ANT and AG sets were merged and four drug-likeness parameters were calculated with DataWarrior: MW, logP, HBD and HBA. Further, a set of molecules was downloaded from ChEMBL, containing structures with MW values ranging between 226.3 and 796.6 g/mol, logP values between 0.837 and 11.074, 1–12 HBA and 0–4 HBD atoms. Among these structures, 500 diverse molecules, presumably inactive on TRPV1, were retrieved as the decoy set (DCY). Candidates for repurposing were retrieved from DrugBank database and contained a total of 1981 approved drugs.

In order to train the predictive models, antagonists were considered true positives, and non-antagonists (agonists and inactive molecules) were considered true negatives when predicting antagonists, while agonists were labeled as true positives and non-agonists (antagonists and inactive molecules) as true negatives when predicting agonist molecules. This rationale was used in the attempt to build a framework that discriminates between TRPV1 agonists and antagonists.

A total of 1444 1D and 2D molecular descriptors were calculated for all datasets using PaDEL-Descriptor software. 

### 3.2. Activity Scores

The first independent variable that was established in the proposed framework was the average activity score (AAS). This average score was calculated as the arithmetic mean of three scores based on the structural features of TRPV1 antagonists and agonists: Bemis–Murcko structural skeletons, plain ring systems and clustering based on flexophore descriptors. Firstly, a scaffold analysis was performed to extract Bemis–Murcko skeletons and plain ring systems. For each individual BM skeleton, the specific score (BMS, Bemis–Murcko score) was established by calculating the mean activity value (pIC_50_ for antagonists, pEC_50_ for agonists, 0 for inactive molecules) between all compounds that share the same skeleton. The same rationale was applied for plain rings (PRS, plain ring score). However, when PR scores were calculated for each compound, we took into account that some molecules contain more than one ring. Thus, for compounds with multiple rings, the mean PR score was calculated as the arithmetic mean between scores of all rings. Moreover, if one ring is present more than once in a structure (e.g., phenyl radical), then the score was calculated considering only one apparition of that ring. Lastly, the similarity cluster score (SCS, flexophore cluster score) was established by calculating the mean activity values between compounds that fall into one specific cluster. Therefore, two average activity scores were calculated, one for predicting antagonists (Score-ANT), and another for agonists (Score-AG). 

After performing the scaffold analysis, a total of 591 BM structures resulted from the group of 3564 compounds (antagonists, agonists and inactive molecules). Most of the scaffolds are formed by 3 or 4 cyclic structures. For each BM skeleton the corresponding ANT and AG scores were calculated. For a total of 256 skeletons, both scores were zero. For the remaining 335 structures, we analyzed the relationship between the two scores (Figure S1A). Interestingly, one BM scaffold ({2-[10-(4-cyclohexylbutyl)-hexadecahydro-1H-indeno[5,4-e]azulen-3b-yl]ethyl}cyclohexane) had both scores above 4, scoring high for both agonist and antagonist prediction. Moreover, 9 agonists and 26 inactive molecules did not contain any rings in their structure and BM skeletons could not be generated.

The PR analysis resulted in 271 unique rings. The most frequent ring is benzene, and it is present in 2682 compounds. Pyridine, the second most frequent ring, is present in 1207 derivatives. The structures contain between 3 and 25 heavy atoms. The corresponding ANT and AG scores were calculated for each PR structure. For a total of 34 rings both scores were zero, and the relationship between the two scores is shown in Figure S1B. Notably, four plain rings had activity scores between 2 and 4, showing less specificity for one activity type: (7aR,11bR,13aR,13bS)-icosahydro-1H-cyclopenta[a]chrysene, (2S,10R,11S)-12,14-dioxatetracyclo[8.7.0.0²,⁶.0¹¹,¹⁵]heptadeca-3,8-dien-5-one, (1S,2R,10S,11R)-12,14,18-trioxapentacyclo[11.4.1.0¹,¹⁰.0²,⁶.0¹¹,¹⁵]octadeca-3,8-dien-5-one and 1,2-dihydroisoquinolin-1-one.

Flexophore descriptors were generated for the merged dataset containing TRPV1 antagonists, agonists and inactive compounds. A total number of 419 clusters was obtained, using a similarity threshold of 80% between flexophores. Scores based on structural similarity clustering were also calculated since some TRPV1 agonists and inactive molecules are acyclic compounds, thus lacking both BM skeletons and plain rings. As observed in Figure S1C, several clusters had mean activity scores between 1 and 4 for both agonist and antagonist prediction (SCS-AG and SCS-ANT), thus being non-specific for either class. A map of flexophore-based similarity relationships generated using automatically determined similarity limits is shown in Figure S1D, highlighting that several TRPV1 antagonists share structural similarity with agonists or experimentally determined inactive compounds. 

The top five scoring BM and PR structures, ranked by activity scores are highlighted in Table 1, for both antagonists and agonists. BM and PR structures were labeled by frequency of apparition in the dataset, in descending order. BM-75 (3-[(decahydronaphthalen-1-yl)methyl]-1,1′-bi(cyclohexane)) and BM-230 (1-[(3,4-dicyclohexylcyclopentyl)methyl]-decahydronaphthalene) are two similar antagonist-specific Bemis–Murcko skeletons with high activity scores, BM-230 having a cyclopentane scaffold instead of cyclohexane, which also has one additional substitution forming an uncondensed tricyclic substructure instead of the bicyclohexane scaffold. In the case of agonists, BM-145 ({2-[10-(3-cyclohexylpropyl)-hexadecahydro-1H-indeno[5,4-e]azulen-3b-yl]ethyl}cyclohexane) and BM-106 ({2-[10-(5-cyclohexylpentyl)-hexadecahydro-1H-indeno[5,4-e]azulen-3b-yl]ethyl}cyclohexane) have highly similar structural skeletons and activity scores, the only difference between the structures being the number of linker atoms (three for BM-145 and five for BM-106). Additionally, structures of BM-264 ((11-cyclopropylundecyl)cyclohexane), BM-176 ((10-cyclopropyldecyl)cyclohexane) and BM-205 ((3-cyclopropylpropyl)cyclohexane) are comprised of two cyclic substructures (cyclohexane and cyclopropane) linked together by an aliphatic chain, the activity score increasing with the number of linker atoms (3 for BM-205, 10 for BM-176 and 11 for BM-264).

Interestingly, the top five scoring plain rings for antagonists are nitrogen heterocycles. Among these fragments, PR-126 (pteridine), PR-127 (pyrido[3,2-d]pyrimidine) and PR-87 (pyrido[2,3-d]pyrimidine) are variations of the same scaffold. Furthermore, azocane (PR-100) had a higher activity score than 2,3,4,5-tetrahydro-1H-2-benzazepine (PR-136), the latter being specific to TRPV1 antagonist CPZ and other related derivatives. The PR with the highest score for agonists (PR-126, 1,2-dihydroisoquinolin-1-one) share structural similarities with the highly ranking antagonist-specific PR-128 (1,2-dihydroquinoxalin-2-one). Moreover, two out of the top five scoring agonist-specific plain rings, PR-57 ((2S,10R,11R)-12,14-dioxatetracyclo[8.7.0.0²,⁶.0¹¹,¹⁵]heptadeca-3,8-dien-5-one) and PR-45 ((1S,2R,10S,11R)-12,14,18-trioxapentacyclo[11.4.1.0¹,¹⁰.0²,⁶.0¹¹,¹⁵]octadeca-3,8-dien-5-one), are also highly similar.

The distributions of activity scores for antagonists, agonists and inactive compounds are shown in Figure S2. As observed, antagonists had overall higher AAS values than agonists, since antagonists represented a significantly larger population among the biologically active compounds. 

The predictive power of the established average activity score was assessed by generating ROC curves and calculating ROC AUC values. The ROC AUC value for antagonist activity scores was 0.963 (Figure S3A), while the same parameter was 0.986 for predicting agonists (Figure S3B), denoting high predictive accuracies in both cases.

### 3.3. Binary Classification

ROC curves were generated based on activity classes and molecular descriptors in order to build classification models using cutoff values. We chose to include a minimum of three and a maximum of eight molecular descriptors as independent variables. ROC AUC values were calculated for all descriptors to assess the discriminatory power of each variable. The eight descriptors were chosen based on four criteria: satisfactory ROC AUC values, statistically significant differences between values of active and inactive molecules, correlation coefficients between each pair of descriptors lower than 0.75 and ease of describing the respective molecular property. 

The selected molecular descriptors and their classification performance parameters are presented in Table 2 and Table 3. The individual ROC curves that were used for choosing the descriptor cutoff values are shown in Figure S4. 

We chose to further discuss some of the selected molecular descriptors for AMG-517 (N-[4-[6-[4-(trifluoromethyl)phenyl]pyrimidin-4-yl]oxy-1,3-benzothiazol-2-yl]acetamide), a potent selective TRPV1 antagonist. Judging by the chosen features, TRPV1 antagonists have secondary nitrogen atoms in their molecules, between 0 and 8 halogen atoms, between 0 and 4 hexa-atomic heterocycles, and 1–15 hydrogen bond acceptors. AMG-517 (Figure S5) has in its structure one secondary nitrogen (substituted amide), three halogen atoms (fluorine), one hexa-atomic heterocycle (pyrimidine), and nine hydrogen bond acceptors (three fluorine atoms, four nitrogens, two oxygens). Moreover, AMG-517 respects seven out of the eight criteria for descriptor threshold values, the only unsatisfied feature being the minimum sum of atom-type E-State for secondary nitrogens. Three of the established criteria require the presence of at least two halogen atoms, at least one hexa-atomic heterocycle, and a minimum five hydrogen bond acceptors.

In the case of TRPV1 agonists, it can be noted that active molecules have 0–3 hydroxyl groups in their molecules, logP values between 0.86 and 10.93, hybridization rations between 0 and 0.95, 0–3 halogen atoms, 0–5 nitrogen atoms, average molecular weight between 5.25 and 9.00, 0–22 atoms in the longest aliphatic chain, and between 3 and 21 atoms in the largest pi system. The established criteria for agonists classification were logP values above 3.023, hybridization ratios higher than 0.383, at least one hydroxyl group, at least 3 atoms in the longest aliphatic chain, the absence of halogen atoms, less than 10 atoms in the largest pi system, less than 3 nitrogen atoms and an average molecular weight lower than 7.25. For instance, the well-known TRPV1 agonist capsaicin ((E)-N-[(4-hydroxy-3-methoxyphenyl)methyl]-8-methylnon-6-enamide) respected all the threshold criteria, having a logP value of 3.983, a hybridization ratio of 0.5, one hydroxyl group, nine atoms in the longest aliphatic chain, no halogen atoms, eight atoms in the largest pi system, one nitrogen atom, and an average molecular weight of 6.228 (Figure S5).

**Figure 3:**
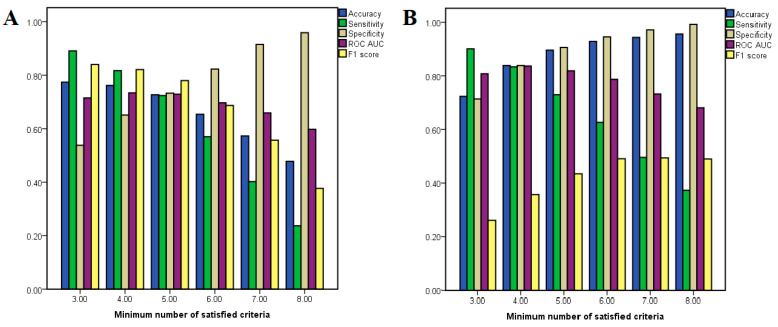
Performance metrics for binary classification in relation to variation of the minimum number of satisfied criteria (molecular descriptor thresholds); (**A**) performance metrics for antagonist classification; (**B**) performance metrics for agonist classification.

Notably, neither AMG-517 nor capsaicin met any of the required criteria for being classified as the opposite type of active compound, and only one molecular descriptor was included in classification models of both agonists and antagonists (number of halogen atoms). The optimal number of required criteria for considering a molecule either an antagonist or an agonist in our classification problem was established by calculating the performance metrics (such as accuracy and ROC AUC) after varying the minimum number of satisfied molecular descriptor thresholds from three to eight. The performance metrics for each classification condition are shown in Figure 3. In the case of antagonists, it can be noted that the model accuracy decreased when the minimum number of required criteria is increased, while in the case of agonists the accuracy varies in proportion to the number of required criteria. 

For classifying antagonists, the accuracy varied between 47.8 and 77.4%, while for agonist classification models the same parameter ranged between 72.5 and 95.8%. The classification model for predicting antagonists with the most optimal balance between sensitivity (0.724) and specificity (0.733) had a minimum number of five satisfied criteria, the accuracy of the model being 72.7%, showing an ROC AUC value of 0.729. On the other hand, the most balanced model for classifying agonists had a minimum required criteria of four satisfied descriptor thresholds, the model’s accuracy being 83.9%, showing sensitivity and specificity values of 0.834 and 0.839, respectively and an ROC AUC of 0.837. Although the model that used a minimum number of five criteria had better accuracy (89.7%), the ROC AUC value was lower (0.819) and this classification model showed a higher preference for true negatives over true positives.

### 3.4. Molecular Docking Simulations

Molecular docking studies were carried out to estimate the binding affinities of potential repurposing candidates and to investigate the predicted binding modes. Molecular docking results were the third and final independent variable in the proposed repurposing predictive model. Two crystal structures were used in this study: activated TRPV1 bound to agonist RTX and TRPV1 in a closed state bound to antagonist CPZ. Both qualitative and quantitative validations of the docking procedure were performed. First of all, the accuracy of binding mode predictions was assessed by docking the co-crystallized ligands into the binding site and superposing the predicted conformation with the experimentally determined ligand pose. The RMSD values calculated after superposition were 1.1277 Å for CPZ and 1.2564 Å for RTX, showing low deviations from original conformation and satisfying accuracy for pose prediction (Figure S6). Binding energies for the positive controls were −9.13 kcal/mol for CPZ and −11.55 kcal/mol for RTX, respectively.

A second validation of the docking protocol was performed by assessing the capability of the two TRPV1 conformations to discriminate against active and inactive ligands by analyzing the predicted binding energies or ligand efficiencies. Therefore, a selection of TRPV1 agonists (n = 194), antagonists (n = 222), inactive molecules (n = 488) and decoys (n = 500) were docked against the binding sites of active (PDB ID 7MZC) and inactive (PDB ID 5IS0) conformations of TRPV1. Molecular docking simulations on TRPV1 in closed conformation yielded binding energies ranging from −13.04 to −5.93 (−9.56 ± 1.096) kcal/mol for antagonists, −11.48 to −5.80 (−8.27 ± 1.260) kcal/mol for agonists, −11.05 to −3.39 (−8.12 ± 1.213) kcal/mol for inactive ligands and from −11.13 to −4.78 (−7.54 ± 1.171) for decoys. The differences in binding energies between antagonists and inactive molecules, and between antagonists and decoys, were statistically different (*p* < 0.05, Student’s independent *t*-test).

After docking on the open state receptor conformation, binding energies between −12.21 and −5.61 (−8.51 ± 1.412) kcal/mol were obtained for agonists, −12.15 and −5.62 (−9.20 ± 0.934) kcal/mol for antagonists, −11.48 and −3.92 (−8.13 ± 1.187) kcal/mol for inactive compounds and between −11.53 and −4.77 (−7.52 ± 1.088) kcal/mol for decoys. Although statistically significant differences were observed between the binding energies of agonists and inactive molecules and between the docking scores of agonists and decoys (*p* < 0.05, independent *t*-test), the differences were not strong enough to discriminate well between agonists and experimentally determined inactives. Due to this inconvenience, a derived parameter was calculated in order to solve the issue of comparable docking scores between agonists and inactive ligands. Thus, the ligand efficiency-dependent lipophilicity index (LELP) was calculated for all the docked ligands, which is expressed as logP divided by ligand efficiency. Mean LELP values were obtained as follows: 13.44 for antagonists, 18.90 for agonists, 9.40 for inactive molecules and 9.61 for decoys. Using LELP values, statistically significant differences were observed between agonists and inactive ligands and between agonists and decoys, respectively (*p* < 0.05). 

**Figure 4:**
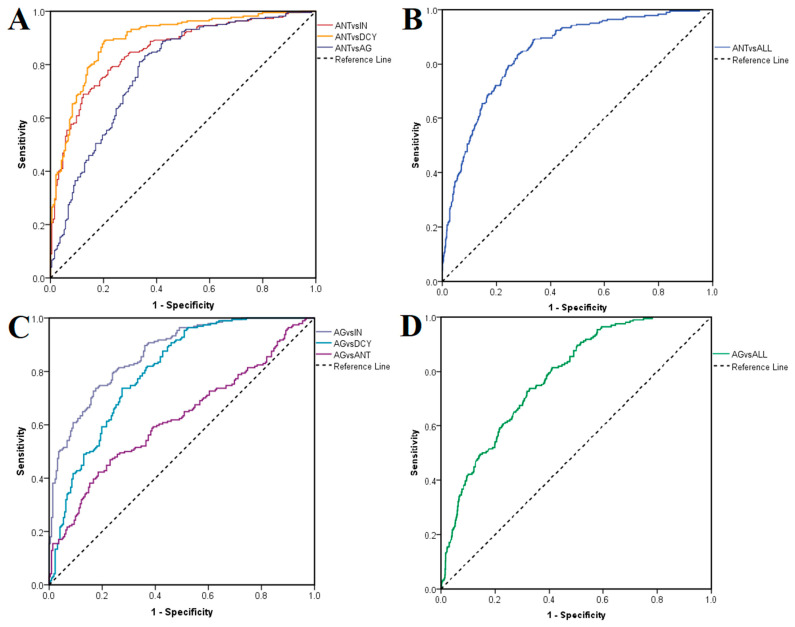
ROC curves showing prioritization of active ligands over inactive compounds and decoys. (**A**) Prioritization of antagonists over inactive compounds, decoys and agonists; (**B**) prioritization of antagonists over all non-antagonists; (**C**) prioritization of agonists over inactive compounds, decoys and antagonists; (**D**) prioritization of agonists over all non-agonists.

ROC curves were generated to assess the suitability of the docking procedure for discriminating between active and inactive ligands. In the first case, antagonists were labeled as positives and non-antagonists (agonists, inactive compounds and decoys) as negatives after docking on the closed conformation of TRPV1. ROC AUC values of 0.852 were obtained after testing antagonists against inactive ligands, 0.894 against decoys, 0.779 against agonists (Figure 4A) and 0.844 against all non-antagonists (Figure 4B). The same rationale was applied after docking on the open conformation, when agonists were treated as positives and non-agonists as negatives, LELP being used in this case instead of binding energy as a classifier. ROC AUC values were 0.868, 0.799 and 0.623 when testing agonists against inactive compounds, decoys and antagonists (Figure 4C), and 0.780 when testing against all non-agonists, respectively (Figure 4D). Thus, the best performance was observed when inactive ligands were treated as negatives. Notably, the molecular docking experiment showed greater accuracies in predicting true antagonists than true agonists. 

### 3.5. Integrated Predictive Model Based on Neural Networks

After establishing activity scores, the number of satisfied descriptor criteria, and binding affinities and efficacies for antagonists, agonists and inactive molecules, these data were integrated into one global predictive model in order to increase the predictive accuracy by adding weights to each of the aforementioned parameters. Since antagonist, agonist and inactive datasets are rather unbalanced, we generated the machine learning model using only the compounds that were selected for molecular docking, thus creating a more balanced training dataset. The machine learning algorithm that we selected for this task was the multilayer perceptron neural network since it also allows the prediction of multiple classes. The architecture with the most optimal parameters had the following characteristics: six input nodes (average activity scores and satisfied descriptor criteria for both antagonists and agonists, binding energies for antagonists, LELP values for agonists), one hidden layer with four neurons (which, in fact, represents the geometric mean between the number of input and output nodes) activated with tanh function, and the output layer with three nodes corresponding to the probabilities for each of the three classes, generated with the softmax function. The most optimal values for the hyperparameters were 0.5 for initial learning rate, 0.7 for momentum and 25 for maximum number of epochs.

**Figure 5:**
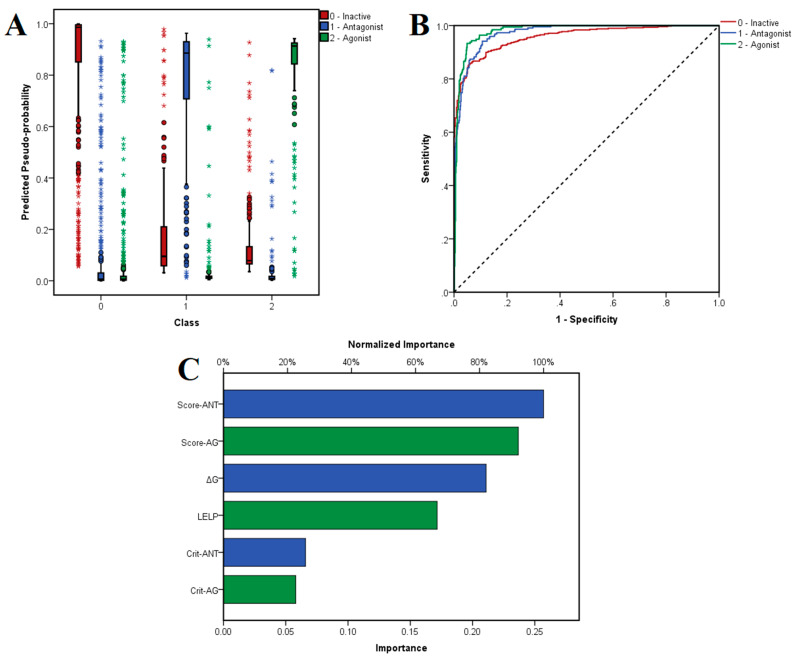
(**A**) Estimated pseudo-probabilities for experimentally determined TRPV1 antagonist, agonists and inactive molecules after applying the MLP NN algorithm; (**B**) ROC curves for classifying TRPV1 antagonists, agonists and inactive molecules; (**C**) importance metrics for independent variables used in the multi-class classification model; Crit-ANT—number of satisfied molecular descriptor criteria for predicting antagonists, Crit-AG—number of satisfied molecular descriptor criteria for predicting agonists.

The chosen neural network correctly predicted 86.3% of inactive molecules, 86.2% of antagonists and 89.4% of antagonists in the training set, with an overall 86.9% prediction accuracy. Moreover, the trained model did not suffer from overfitting, since the algorithm correctly predicted 90.7% of the ligands from the test set: 89.7% of inactive compounds, 90.0% of antagonists and 94.3% of agonists. The distribution of the predicted pseudo-probabilities is shown in Figure 5A. The overall prediction accuracy was 88.8% for predicting inactive compounds, 92.04% for antagonists and 93.70% for agonists, the model showing better accuracies for correctly predicting agonists and antagonists (Table 4). ROC AUC values were 0.957 for classifying inactive ligands, 0.972 for antagonists and 0.980 for agonists (Figure 5B). The generated classification model showed higher values for specificity over sensitivity, and thus the algorithm identifies true negatives relatively more accurately than true positives.

The independent variables with the highest importance in predicting the three classes were average activity scores for antagonists and agonists, followed by binding energy and LELP, while the numbers of satisfied molecular descriptor criteria for antagonists and agonists had the lowest weights (Figure 5C). 

### 3.6. Prediction of Potential TRPV1 Modulators through Drug Repurposing

The 1981 approved drugs retrieved from the DrugBank database were subjected to the virtual screening protocol to identify potentially new TRPV1 modulators. Firstly, graph mining techniques were used with DataWarrior software to append appropriate activity scores for the repurposing molecules. Then, scaffold analysis and flexophore similarity search were performed to retrieve Bemis–Murcko, plain rings and cluster similarity scores for approved drugs that had similar structural features with experimentally determined TRPV1 antagonists, agonists and inactive molecules. After performing the analysis, average activity scores varied between 0 and 6.23 (1.42 ± 1.151) for predicting antagonists and between 0 and 4.40 (0.41 ± 0.549) for predicting agonists. Only five compounds had higher activity scores than the mean score of antagonists (5.79) and only one drug showed a higher activity score than the mean value for agonists (3.01).

Among the screened compounds, 686 (34.6%) have in their structure Bemis–Murcko skeletons present among TRPV1 antagonists, and 725 (36.6%) molecules have BM skeletons observed within agonists. A total of 1348 (68%) compounds contain plain rings contained by structures of antagonists, and 1406 (80%) drugs contain plain rings commonly found within agonists, which denotes that agonists contain aromatic or non-aromatic rings that are not highly specific to TRPV1 agonistic activity. After performing the similarity search based on flexophore descriptors, we found that 333 (16.8%) and 146 (7.4%) approved drugs shared structural similarities higher than 0.8 with antagonists and agonists, respectively.

Furthermore, 1D and 2D molecular descriptors were generated for marketed drugs to establish the number of relevant molecular features that are shared with known TRPV1 ligands. We previously showed that a compound should satisfy at least five antagonist-specific molecular descriptor criteria to be classified as an antagonist and at least four agonist-specific criteria to be considered an agonist, with a good balance between sensitivity and specificity. After applying the thresholds for the 16 chosen molecular descriptors, 315 (15.9%) molecules satisfied the condition of being classified as antagonists, and 1121 (56.6%) compounds met the criteria for being considered potential agonists. These results hinted toward the fact that the minimum required number of descriptor criteria for classifying agonists is too permissive. On another note, we observed while training the neural networks that the machine learning algorithm performed more accurately when we used the total number of the satisfied descriptor criteria instead of the binary categorical values (1 for active antagonist or agonist, 0 for inactive) as classifiers. The last two observations led to the treatment of the molecular descriptor-derived property as an ordinal independent variable, rather than dichotomous. 

Molecular docking experiments were carried out to estimate binding energies after predicting the interaction with a closed-state conformation of TRPV1, and the ligand efficiency-dependent lipophilicity index after simulating interactions with the agonist-bound conformation of the ion channel. Among the screened drugs, 79 (4%) molecules showed lower binding energies than the mean value for TRPV1 antagonists (−9.56 kcal/mol) after docking into the antagonist-specific binding site. After simulating the interactions with the agonist-specific conformation of the binding site, 276 (13.9%) compounds had lower energies than the mean value for agonists (−8.51 kcal/mol), and 147 (7.4%) showed LELP values higher than the mean for agonists (18.90).

The distribution of the established input variables for class prediction is depicted in Figure S7. As observed, only the number of satisfied descriptor criteria for predicting agonists, predicted binding energies and calculated LELP followed normal distributions among the screened drugs.

**Figure 6:**
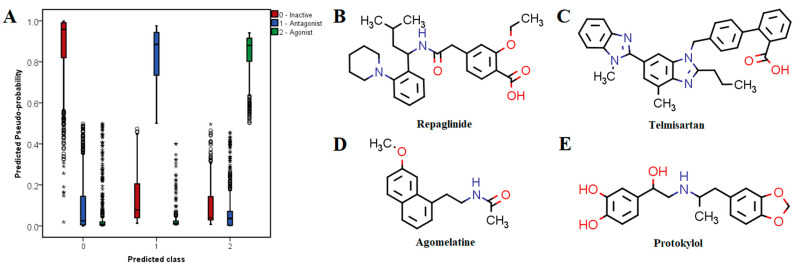
(**A**) Distribution of multi-class predicted probabilities using neural networks; (**B**–**D**) proposed drug repurposing candidates predicted as TRPV1 antagonists; (**E**) proposed drug repurposing candidate predicted as TRPV1 agonists.

The independent variables that were determined after performing the prerequisite screening were fed into the validated neural network. The distribution of the estimated probabilities corresponding to each of the predicted classes yielded after applying the integrated machine learning model is illustrated in Figure 6A. The implemented model predicted 1112 (56.1%) compounds as inactive molecules, 258 (13%) as potential antagonists and 572 (28.9%) as potential agonists, if a 50% probability threshold was applied for being considered a positive. Moreover, 116 (5.9%) drugs from the predicted antagonist class and 210 (10.6%) from the predicted agonist class showed probabilities over 90%.

The top 10 (0.5%) predicted antagonists ranked by probabilities and their established pharmacological activity are shown in Table 5, while the top 10 predicted agonists are shown in Table 6. Approved drugs with high probabilities of being active antagonists are very structurally and pharmacologically diverse. For instance, the top 10 predicted antagonists have entirely different therapeutic indications and had calculated probabilities over 95%. The top three potential antagonists were repaglinide (antihyperglycemic agent, ATP-dependent potassium channel blocker), telmisartan (antihypertensive, angiotensin II receptor blocker) and tafenoquine (antimalarial agent).

Some of the drugs with high predicted probabilities for acting as TRPV1 agonists were adrenergic receptor modulators, either sympathomimetics (e.g., protokylol, ephedrine, etilephrine and formoterol) or β-blockers (e.g., bisoprolol, esmolol, practolol, celiprolol, sotalol and labetalol), indicating that these molecules share common features with TRPV1 agonists. However, timolol, nadolol, carteolol and prazosin were predicted as potential antagonists. Lidocaine and other local anesthetics (e.g., butacaine, prilocaine, oxetacaine and procaine) were also identified as potential agonists. Calcitriol (active form of vitamin D) showed the highest probability of exerting TRPV1 agonist activity. Moreover, vitamin A and its derivatives (alitretinoin, isotretinoin) were identified among the top 10 potential agonists. Most of these compounds have high lipophilicity, which is a common property for TRPV1 agonists as seen from molecular descriptor analysis and LELP values.

The most promising candidates for repurposing as TRPV1 modulators with pharmacotherapeutic utility in pain relief were chosen based on three criteria: high probability of being active, favorable interactions with relevant residues within the binding site of TRPV1 and acceptable safety profiles. Therefore, three potential antagonists (repaglinide, telmisartan and agomelatine) and one potential agonist (protokylol) were proposed as repositioning candidates and were discussed in further detail (Figure 6B–E).

Repaglinide, an antidiabetic drug acting as a blocker of ATP-dependent potassium channels [54], showed the highest probability of blocking TRPV1 and had an average activity score of 5.42, its structure being characterized by a BM-24 skeleton (2-(4-cyclohexylbutyl)-1,1′-bi(cyclohexane)). Moreover, repaglinide contains in its molecule two phenyl rings (PR-1) and a piperidine scaffold (PR-5), both being present in structures of TRPV1 antagonists. Repaglinide also has a flexophore similarity of 87% with TRPV1 antagonist CHEMBL1779679. Furthermore, repaglinide satisfied three out of eight proposed molecular descriptor criteria, having in its molecule one secondary nitrogen, one hexa-atomic ring containing heteroatoms and six hydrogen bond acceptors. Telmisartan, an antihypertensive agent that blocks angiotensin II receptors [55] showed an average activity score of 4.29, having also the second highest predicted probability of blocking TRPV1. Its structure contained no antagonist-specific BM scaffold, but the benzodiazole (PR-21) and benzene (PR-1) rings had high contributions to the overall score. Moreover, telmisartan had a flexophore similarity of 80.5% with TRPV1 antagonist CHEMBL3961718. Telmisartan satisfied only two molecular descriptor criteria, the measure of electronegative atom count relative to molecular size and molecular complexity. Another interesting potential TRPV1 antagonist was agomelatine, a melatonin naphthalene analog used to treat depressive disorders [56], which had an estimated probability of binding to the channel of 80.25%. This atypical antidepressant had an average activity score of 4.00, its structure being derived from the decahydronaphthalene BM skeleton (BM-90) or naphthalene scaffold (PR-16), which are present among potent TRPV1 antagonists. Agomelatine had 83.1% flexophore similarity with TRPV1 antagonist CHEMBL400371. In fact, the discovered flexophore similarity pair is characterized by strikingly similar structures, since the two molecules share a large structural fragment ((7-methoxynaphthalen-1-yl)ethyl), while the acetamide moiety from agomelatine is replaced with (trifluoromethoxy)benzamide in the TRPV1 antagonist. Although agomelatine contains a secondary nitrogen, its structure did not satisfy any molecular descriptor criteria for antagonists. 

Bronchodilator protokylol [57] showed the second highest estimated probability for acting as a TRPV1 agonist. Protokylol had an average activity score of 2.76, its molecule containing the 5-(5-cyclohexylpentyl)-octahydro-1H-indene BM skeleton (BM-126), represented by the benzene (PR-1) and benzodioxole (PR-44) plain rings, these structural features being also present among TRPV1 agonists. A flexophore similarity match of 87.8% was observed between protokylol and nylidrin. Nylidrin (buphenine) is a β2-adrenoreceptor agonist acting as a vasodilator (withdrawn for lack of effectiveness) [58], which has 15.85 µm potency for TRPV1 agonist activity (pEC_50_ = 4.8 M), the result being declared inconclusive in the ChEMBL database. Protokylol met six out of eight molecular descriptor criteria for acting as a TRPV1 agonist, the only violated thresholds being for XlogP and hybridization ratio.

**Figure 7:**
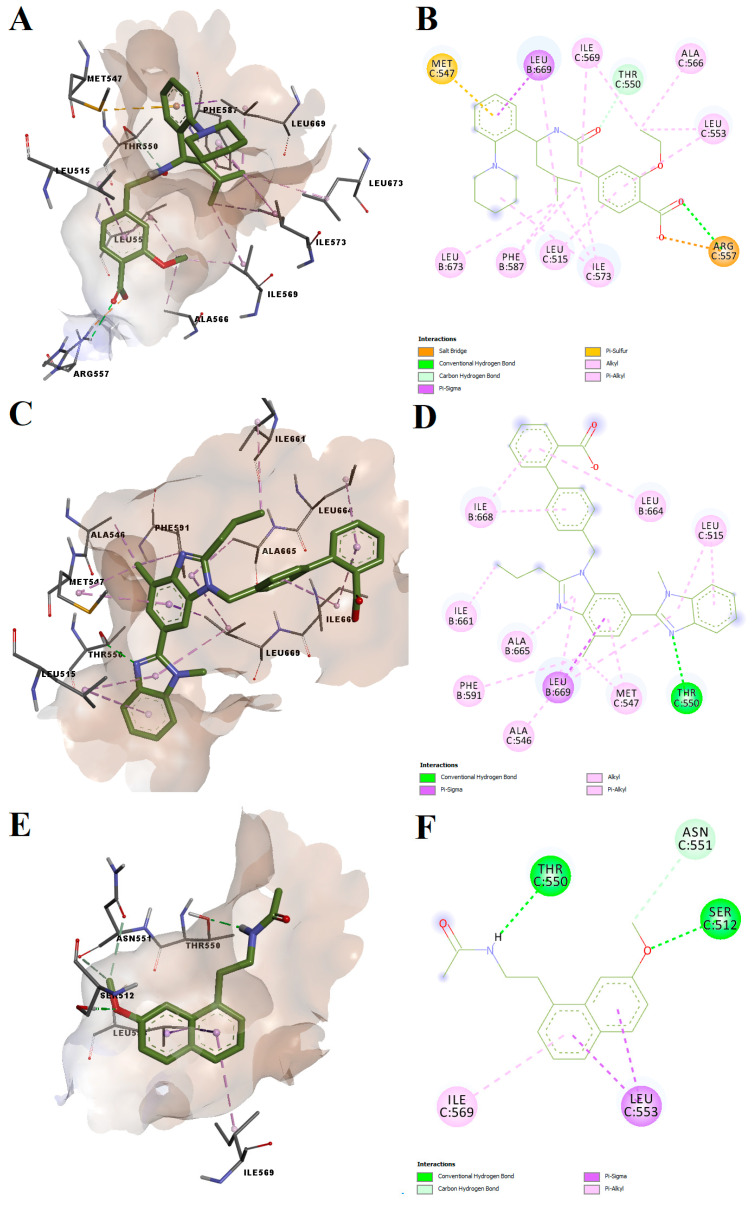
Predicted molecular interactions between potential antagonists and TRPV1 vanilloid binding site. (**A**) Conformation of the predicted repaglinide–TRPV1 complex; (**B**) 2D interaction diagram for the predicted repaglinide-TRPV1 complex; (**C**) conformation of the predicted telmisartan–TRPV1 complex; (**D**) 2D interaction diagram for the predicted telmisartan–TRPV1 complex; (**E**) conformation of the predicted agomelatine–TRPV1 complex; (**F**) 2D interaction diagram for the predicted agomelatine–TRPV1 complex; van der Waals contacts were not displayed for simplicity.

Repaglinide, telmisartan and agomelatine had binding energies after docking into the antagonist-specific conformation of the vanilloid pocket of −7.78, −9.20 and −7.69 kcal/mol, respectively. The predicted conformations of the three potential antagonists were not highly torsioned, while repaglinide and agomelatine adopted spatial orientations similar to antagonist CPZ (Figure 7). All three ligands formed hydrogen bonds or carbon–hydrogen bonds with Thr550, located in the loop between TM4 and 5, which was shown to be a key residue for TRPV1 modulation [32,33]. Moreover, agomelatine forms a hydrogen bond with Ser512 from the loop connecting TM2 and TM3, while repaglinide forms a hydrogen bond and a salt bridge with Arg557, a residue that is relevant for CPZ antagonist activity. For instance, CPZ forms hydrogen bonds with Thr550, Ser512 and Arg557. Multiple non-polar interactions were also observed between the docked ligands and residues within the vanilloid binding pocket, the docked ligands showing high potential for blocking the TRPV1 channel by acting on the CPZ binding site. On the other hand, the structure of telmisartan lacks a vanillyl head analogous scaffold and therefore did not bind as deep into the vanilloid pocket as other ligands.

**Figure 8:**
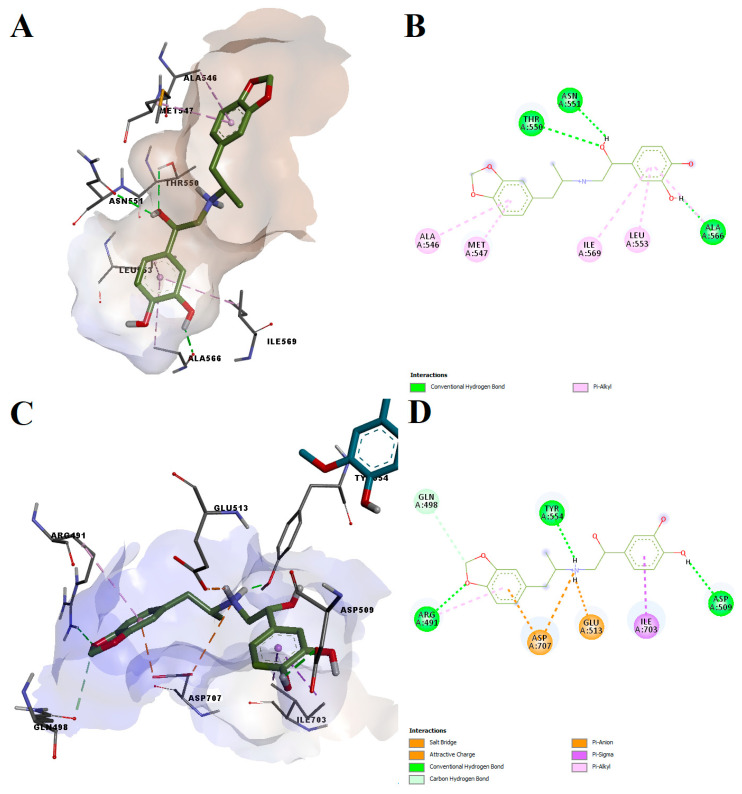
Predicted molecular interactions between protokylol and different overlapping TRPV1 binding sites. (**A**) Conformation of the predicted protokylol–TRPV1 complex after binding to the vanilloid site; (**B**) 2D interaction diagram for the predicted protokylol–TRPV1 complex after binding to the vanilloid site; (**C**) conformation of the predicted protokylol–TRPV1 complex after binding to the phosphoinositides site (fragment of RTX bound to the vanilloid pocket is depicted in blue); (**D**) 2D interaction diagram for the predicted protokylol–TRPV1 complex after binding to the phosphoinositides site; van der Waals contacts were not displayed for simplicity.

Protokylol showed a predicted binding energy of −8.27 kcal/mol after simulating the interaction with the agonist-bound conformation of the TRPV1 active site, the calculated LELP being 5.52. Similar to the other analyzed ligands, protokylol fit very well within the binding cavity (Figure 8A), showing similar orientations with known TRPV1 ligands. Protokylol formed three hydrogen bonds with Thr550, Asn551 and Ala566 and multiple non-polar pi-alkyl interactions with other residues (Figure 8B). Interestingly, after analyzing the third predicted conformation, we found that protokylol could potentially bind to the phosphoinositides-specific binding site (−7.52 kcal/mol, Figure 8C,D). This binding site overlaps with the vanilloid binding pocket and can inhibit capsaicin-induced activation of TRPV1 [59]. Protokylol formed a salt bridge and a pi-anion interaction with Asp707 and a pi-sigma interaction with Ile703, residues that were shown to be essential for TRPV1 modulation by phosphoinositides [59]. Additionally, the protonated secondary amine moiety also interacted with Glu513 through attractive charges. Moreover, three hydrogen bonds and one carbon–hydrogen bond were formed with other residues.

## 4. Discussion

The ligand-dependent cation channel TRPV1 is a promising target for managing various types of pain disorders [3]. In recent years, an increasing number of studies have focused on discovering TRPV1 antagonists and agonists/desensitizers as therapeutic solutions for chronic, neuropathic and inflammatory pain, but also for migraine and cluster headache [8,19,60]. Unfortunately, the approval of TRPV1 antagonists is hindered due to severe side effects observed in clinical trials, such as hyperthermia. However, the discovery of novel TRPV1 modulators with therapeutic utility in pain conditions can be accelerated by channeling efforts into drug repurposing approaches, including consecrated computer-aided drug discovery methods. Drug repositioning strategies are advantageous due to the significantly lower human, financial and temporal resources required for discovering new therapies for diseases that lack optimal therapeutic options, by identifying new pharmacological actions for marketed drugs with well-established pharmaco-toxicological profiles [36]

In our study, we focused on implementing an in silico drug repurposing framework aimed at predicting novel TRPV1 antagonists and agonists/desensitizers with analgesic activity. The predictive model was developed to discriminate between TRPV1 antagonists and agonists since virtual screening approaches can lead to discovering ligands with activity types opposite to the predicted outcome. For instance, in a previous work, we identified febuxostat (xanthine oxidase inhibitor) as a potential TRPA1 antagonist [48], which was proven thereafter to act as a weak TRPA1 agonist [61]. Both ligand-based and structure-based approaches were applied to generate a set of variables that were further used to develop an artificial intelligence algorithm based on neural networks aimed at predicting the probabilities of tested molecules behaving as TRPV1 antagonists, agonists/desensitizers, or being classified as inactive. Artificial neural networks were chosen as an end-point predictive model due to their increasing usefulness in drug discovery campaigns. However, neural networks are often elusive in terms of comprehensibility of nonlinear relationships between chemical structural features and biological activity, and other authors have suggested that predictive accuracy could be sacrificed in favor of highly interpretable models [62]. 

Foremost, this paper presents our strategy for implementing a relative sense of linearity in characterizing the relationships between the physicochemical and structural properties of TRPV1 modulators and the biological outcome. In this regard, activity scores based on biological activity measures were assigned to structural scaffolds of known TRPV1 active and inactive molecules, such as Bemis–Murcko skeletons and plain rings, to transform the building blocks present within molecules of active TRPV1 modulators into continuous variables. Therefore, structural scaffolds that yield highly potent TRPV1 ligands were identified. For instance, one such substructure was the pyrido[2,3-d]pyrimidine heterocycle, which is considered a privileged scaffold that can provide compounds with diverse pharmacotherapeutic indications (e.g., antihypertensive, antidiabetic and antitumor agents) [63]. Further, structural flexibility and pharmacophoric characteristics were also transformed, by assigning activity scores based on clustering using a flexophore descriptors similarity threshold of 80%. The calculated average activity score was proven highly accurate in discriminating between active and inactive molecules.

Several molecular descriptors were chosen for our predictive model by analyzing ROC curves and relevance in characterizing TRPV1 ligands. In this case, we established a set of rules defined as certain thresholds for descriptor values that should be respected to confer antagonist or agonist activity on TRPV1 receptors. Hence, an ordinal categorical variable was defined, which illustrates the number of such criteria satisfied by each molecule (from three to eight). The classification model based on these rules was proven to be more accurate in correctly classifying known agonists than antagonists since the antagonist dataset is considerably more structurally diverse. However, the models generated for classifying agonists had a relatively lower F1 score (weighted average of precision and recall), possibly due to a more unbalanced dataset (agonists/non-agonists ratio of 1:17). This issue was further addressed in the process of training the neural networks, by pruning the antagonists and inactives datasets based on chemical similarity, yielding a training dataset consisting of an approximately 1:1:2 ratio of agonist/antagonist/inactive compounds.

Molecular docking simulations were carried out using agonist- and antagonist-bound TRPV1 channel conformations. The docking procedure was more successful in classifying antagonists. The resulting binding energies and LELP values were further used as variables in training the neural networks. 

Independent variables obtained from the scaffold and flexophore analysis established molecular descriptors criteria and molecular docking screening were used to train the machine learning model. The developed multilayer perceptron neural network algorithm showed higher accuracies in correctly predicting antagonists and agonists than inactive molecules. On the other hand, specificity values were considerably higher for all three classes. These observations translate into the fact that the proposed model behaves more accurately when assigning molecules to antagonist and agonist classes, while there is a smaller chance that the algorithm would predict false positives. The neural network architecture also attenuated the modest predictive performances of molecular descriptors and docking on agonist-specific conformation by assigning lower weights to the variables described as the number of satisfied descriptor criteria and LELP.

The dataset containing the approved drugs retrieved from the DrugBank database was subjected to the same analysis as TRPV1 ligands in order to predict probabilities of acting on the TRPV1 receptor by using the developed neural network. As observed in many drug discovery campaigns, docking scores alone do not often yield remarkably high success rates in discovering true positives [53] or appropriate repurposing candidates. For instance, among the top 1% (20) molecules ranked by predicted binding affinities, only 6 showed antagonist activity probabilities higher than 0.5. Moreover, antitumoral tyrosine kinase inhibitors (nilotinib, sorafenib, capmatinib and imatinib) were noticed among the top-scoring drugs ranked by binding affinity, which cannot be repurposed as analgesics due to high toxicities. Interestingly, despite having a very high predicted binding affinity to TRPV1 (−10.52 kcal/mol), paliperidone was predicted as inactive, which is in concordance with previously published molecular biology studies that assessed the interaction with TRPV1 activity [61]. Overall, the screened compounds with a high probability of acting as TRPV1 antagonists are highly pharmacologically diverse. The first two molecules ranked by TRPV1 inhibition probability were the antidiabetic repaglinide and the antihypertensive telmisartan.

Binding pose analysis of repaglinide revealed its potential to competitively block the vanilloid binding site of TRPV1. Moreover, our analysis showed that repaglinide shares relevant structural features with certain TRPV1 antagonists. To the best of our knowledge, there are no available preclinical studies that analyzed the analgesic potential of repaglinide. Frequent adverse reactions such as hypoglycemia and weight gain associated with repaglinide treatment [64] indicate that its use as an analgesic agent might be limited to patients suffering from diabetic neuropathy. 

The analgesic and anti-inflammatory effects of telmisartan have been evaluated in preclinical studies of different types of neuropathic pain [65,66,67,68]. The main mechanism underlying these effects is partial activation of PPAR-γ receptors [65,66,68], but according to one study, telmisartan reduced thermal and mechanical hypersensitivity by inhibiting CYP2J2 [67]. In addition, Sisignano et al. used electrophysiological measurements and calcium-imaging experiments to investigate the possibility of interaction between telmisartan and the TRPV1 channel and did not observe an effect of the substance on TRVP1-dependent calcium transients or inward currents [67]. Considering these findings, telmisartan can be considered as a false positive discovered by our algorithm as a potential TRPV1 antagonist. The lack of telmisartan activity on TRPV1 could be explained by the molecular docking results. Unlike other ligands, telmisartan lacks a vanilloid-like head substructure and therefore its conformation cannot fit as well into the vanilloid pocket.

Another interesting compound that was predicted as a TRPV1 antagonist was agomelatine, a naphthalene analog of melatonin. Agomelatine fits satisfyingly into the vanilloid binding pocket, and it shares a high similarity with a TRPV1 antagonist, the methyl radical within the acetamide moiety being replaced with the trifluoromethoxy-methyl substructure in the known antagonist. Interestingly, melatonin was shown to regulate intracellular calcium influx, possibly by inhibiting the TRPV1 channel [69]. Since agomelatine is an analog that acts as an agonist of the melatoninergic receptors MT_1_ and MT_2_ [70], other studies investigated whether agomelatine shows a melatonin-like effect on TRPV1 receptor signaling, but direct interaction with the channel was not demonstrated, since no direct and specific experiments were performed to assess the binding of agomelatine to TRPV1 receptor [71]. The role of agomelatine in pain is supported by few preclinical studies [72,73]. In this regard, it has been observed that in various animal models of neuropathic pain (e.g., induced by streptozotocin, oxaliplatin or chronic constriction nerve injury), agomelatine ameliorated pain-associated hypersensitivity [73]. 5-HT_2C_ and MT_2_ receptors are involved in pain modulation [74,75,76,77]. Researchers believe that the analgesic effect of agomelatine is due to its action on these receptors (agonist on melatoninergic receptor and antagonist on the 5-HT_2C_ receptor) [73]. Agomelatine has an optimal safety profile, with few side effects (such as dizziness, nausea, diarrhea and dry mouth) occurring especially early in the treatment [78].

Calcitriol, the active form of vitamin D, had the highest probability of acting as a TRPV1 agonist. Moreover, a recently published study revealed that vitamin D acts as a partial agonist of the TRPV1 channel, this discovery being in line with our prediction [79].

Protokylol, a β2-adrenergic agonist used as a bronchodilator [57] emerged as a compound with the second highest probability of exerting agonist activity on TRPV1. Two different, overlapping binding sites were observed for protokylol, one specific to vanilloids and competitive antagonists and another for phosphoinositides [59]. Protokylol formed favorable interactions with relevant amino acid residues within both binding sites. Therefore, protokylol could potentially show analgesic activity either by desensitizing TRPV1 through interaction with the vanilloid binding site or by inhibiting the channel through allosteric modulation, similar to phosphoinositides [59]. No preclinical studies regarding the analgesic potential of protokylol are available. The main side effects of beta-adrenergic agonists such as protokylol are mild tachycardia, tremor or metabolic effects [80]. Moreover, other adrenergic receptor modulators (bisoprolol, esmolol, practolol, celiprolol, sotalol and labetalol) were also identified by the repurposing algorithm as potential TRPV1 agonists. A previous study revealed that catecholamines can upregulate TRPV1 activity in cultured neurons, but no direct interactions between adrenergic modulators and TRPV1 receptor were studied [81].

Due to their optimal predicted binding into TRPV1 active sites and high estimated probabilities of being active ligands, we propose repaglinide and agomelatine as potential TRPV1 antagonists and protokylol as a potential TRPV1 agonist/desensitizer. Further studies are required to experimentally evaluate the interactions between the proposed molecules and TRPV1 and to investigate their activity in various animal models of pain-related disorders.

## 5. Conclusions

An in silico drug repurposing framework was established using ligand-based, structure-based and machine learning approaches for identifying novel TRPV1 modulators. After screening the approved drugs with the validated algorithm, repaglinide (antidiabetic) and agomelatine (antidepressant) emerged as potential TRPV1 antagonists, and protokylol (bronchodilator) as an agonist. Further studies are required to confirm the predicted activity on TRPV1 and to assess the candidates’ efficacy in alleviating pain.

## Figures and Tables

**Table 1:** Top five Bemis–Murcko skeletons and plain rings ranked by activity scores for both antagonists and agonists.

	Antagonists				Agonists			
Type	Label	Structure	Score	Frequency	Label	Structure	Score	Frequency
Bemis–Murcko skeletons	BM-487	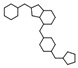	9.00	1 (0.03%)	BM-264	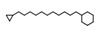	8.50	1 (0.03%)
	BM-433	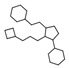	8.90	1 (0.03%)	BM-176	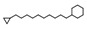	8.04	3 (0.08%)
	BM-176	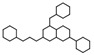	8.84	3 (0.08%)	BM-145	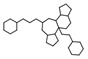	8.04	4 (0.11%)
	BM-75	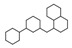	8.84	9 (0.25%)	BM-106	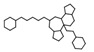	7.96	6 (0.17%)
	BM-230	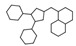	8.79	2 (0.06%)	BM-205	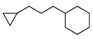	7.65	2 (0.06%)
Plain rings	PR-126	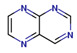	9.68	1 (0.03%)	PR-126	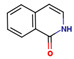	7.36	1 (0.03%)
	PR-127	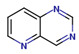	9.20	1 (0.03%)	PR-57	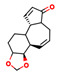	7.36	12 (0.34%)
	PR-128	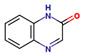	9.15	1 (0.03%)	PR-127	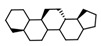	6.90	1 (0.03%)
	PR-87	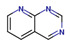	7.98	3 (0.08%)	PR-45	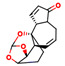	6.15	20 (0.56%)
	PR-100	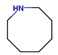	7.94	2 (0.06%)	PR-100	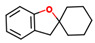	5.53	2 (0.06%)

**Table 2:** Molecular descriptors included in the classification models for antagonist prediction.

Descriptor	Meaning	Threshold	Sensitivity	Specificity	ROC AUC
SssNH	Sum of atom-type E-State: -NH-	>2.962	0.681	0.689	0.743
MDEN-22	Molecular distance edge between all secondary nitrogens	>0.454	0.678	0.685	0.735
ETA_BetaP_s	A measure of electronegative atom count of the molecule relative to molecular size	>0.638	0.676	0.662	0.734
nX	Number of halogen atoms	≥2	0.641	0.730	0.727
fragC	Complexity of a system	>172.070	0.682	0.669	0.724
n6HeteroRing	Number of six-membered rings containing heteroatoms	at least 1	0.783	0.547	0.723
nHBAcc2	Number of hydrogen bond acceptors (any oxygen; any nitrogen where the formal charge of the nitrogen is non-positive, except a non-aromatic nitrogen that is adjacent to an oxygen and aromatic ring, or an aromatic nitrogen with a hydrogen atom in a ring, or an aromatic nitrogen with three neighboring atoms in a ring, or a nitrogen with total bond order ≥4; any fluorine)	>5	0.753	0.574	0.715
maxHother	Maximum atom-type H E-State: H on aromatic CH, =CH_2_ or =CH-	>0.631	0.678	0.678	0.723

**Table 3:** Molecular descriptors included in the classification models for agonist prediction.

Descriptor	Meaning	Threshold	Sensitivity	Specificity	ROC AUC
XLogP	Octanol/water partition coefficient	>3.023	0.689	0.700	0.788
HybRatio	Fraction of sp3 carbons to sp2 carbons	>0.383	0.689	0.719	0.762
nsOH	Count of atom-type E-State: -OH	>0	0.736	0.762	0.761
nAtomLAC	Number of atoms in the longest aliphatic chain	>2	0.668	0.754	0.758
nX	Number of halogen atoms	=0	0.855	0.702	0.806
nAtomP	Number of atoms in the largest pi system	<10	0.627	0.918	0.825
nN	Number of nitrogen atoms	<3	0.870	0.721	0.848
AMW	Average molecular weight (Molecular weight/Total number of atoms)	<7.250	0.751	0.802	0.860

**Table 4:** Performance metrics for the multi-class neural network.

	Class		
Parameter	Inactive	Antagonist	Agonist
Accuracy	0.888	0.920	0.937
Sensitivity	0.867	0.851	0.876
Specificity	0.913	0.943	0.954
ROC AUC	0.957	0.972	0.980
F1 score	0.893	0.840	0.856

**Table 5:** Top 0.5% predicted TRPV1 antagonists ranked by estimated probability.

Rank	DrugBank ID	Generic Name	Pharmacological Class	Score-ANT	Crit-ANT	ΔG (kcal/mol)	Probability (ANT)
1	DB00912	Repaglinide	antidiabetic (meglinide)	5.42	3	−7.78	0.9759
2	DB00966	Telmisartan	antihypertensive (angiotensin II receptor antagonist)	4.29	2	−9.20	0.9730
3	DB06608	Tafenoquine	antiparasitic	4.66	5	−7.53	0.9718
4	DB00836	Loperamide	antidiarrheals (opioid agonist)	3.80	2	−8.59	0.9703
5	DB09056	Amorolfine	antifungal	4.74	1	−8.42	0.9703
6	DB08820	Ivacaftor	CFTR potentiator	3.74	3	−9.34	0.9695
7	DB14677	Gestonorone caproate	progestin medication	4.48	1	−7.15	0.9694
8	DB08976	Floctafenine	NSAID	4.56	5	−7.48	0.9692
9	DB00354	Buclizine	antiallergic (H1 receptor antagonist)	3.70	2	−8.21	0.9680
10	DB06202	Lasofoxifene	selective ER modulator	3.91	1	−7.94	0.9678

Crit-ANT—number of satisfied molecular descriptor criteria for predicting antagonists.

**Table 6:** Top 0.5% predicted TRPV1 agonists ranked by estimated probability.

Rank	DrugBank ID	Generic Name	Pharmacological Class	Score-AG	Crit-AG	ΔG (kcal/mol)	LELP	Probability (AG)
1	DB00136	Calcitriol	active form of vitamin D	2.92	8	−8.27	21.65	0.9416
2	DB06814	Protokylol	adrenergic β2 receptor agonist	2.76	6	−8.71	5.52	0.9412
3	DB00523	Alitretinoin	retinoid (antineoplastic agents)	2.07	6	−7.29	17.56	0.9405
4	DB01597	Cilastatin	dipeptidase inhibitor	2.62	5	−7.29	−2.76	0.9389
5	DB01187	Iophendylate	radiocontrast agent	1.24	5	−6.66	21.40	0.9387
6	DB00162	Vitamin A	retinoid	2.07	7	−8.26	15.68	0.9384
7	DB00982	Isotretinoin	retinoid (anti-acne agent)	2.07	6	−8.79	14.56	0.9375
8	DB11570	Padimate O	sunscreen agent	1.60	6	−7.01	12.46	0.9374
9	DB04822	Oxeladin	antitussive agent	1.24	7	−6.59	13.55	0.9372
10	DB11594	Domiphen	antiseptic	1.24	7	−6.31	12.55	0.9368

Crit-AG—number of satisfied molecular descriptor criteria for predicting agonists; ligand efficiency-dependent lipophilicity index.

## Data Availability

The data presented in this study are available on request from the corresponding authors.

## Supplementary Materials

The following supporting information can be downloaded at: https://www.mdpi.com/article/10.3390/pharmaceutics14122563/s1, Figure S1: (A) Relationship between BM scores for predicting antagonists and agonists; (B) relationship between PR scores for predicting antagonists and agonists; (C) relationship between SC scores for predicting antagonists and agonists (representative structures are highlighted); (D) map of structure similarity relationships based on flexophores for TRPV1 antagonists (class 1), agonists (class 2) and inactive molecules (class 0); Figure S2: Box plots representing activity scores established based on structural features for both antagonists and agonists. (A) Bemis-Murcko activity scores for predicting antagonists (BMS-ANT); (B) Bemis-Murcko activity scores for predicting agonists (BMS-AG); (C) plain rings activity scores for predicting antagonists (PRS-ANT); (D) plain rings activity scores for predicting agonists (PRS-AG); (E) flexophore similarity cluster activity scores for predicting antagonists (SCS-ANT); (F) flexophore similarity cluster activity scores for predicting agonists (SCS-AG); (G) average activity scores for predicting antagonists (Score-ANT); (H) average activity scores for predicting agonists (Score-AG); Figure S3: ROC curves showing predictive capacity of average activity scores. (A) activity scores for predicting antagonists (Score-ANT); (B) activity scores for predicting agonists (Score-AG); Figure S4: ROC curves showing discriminant capacity of each selected descriptor. (A) descriptors included for antagonists classification (larger values indicate more positive test result); (B) descriptors included for agonists classification, with higher values indicating more positive test result; (C) descriptors included for agonists classification, with lower values indicating more positive test result; Figure S5: Chemical structures of TRPV1 antagonist AMG-517 and TRPV1 agonist capsaicin; Figure S6: Validation of binding pose prediction. (A) superposition between predicted (purple) and experimental (green) conformations for CPZ; (B) superposition between predicted (purple) and experimental (green) conformations for RTX; Figure S7: Distribution of input variables established for approved drugs (DrugBank). (A) distribution of average activity scores for predicting antagonists (Score-ANT); (B) distribution of number of satisfied molecular descriptor criteria for predicting antagonists (Crit-ANT); (C) distribution of binding energies for predicting antagonists (ΔG); (D) distribution of average activity scores for predicting agonists (Score-AG); (E) distribution of number of satisfied molecular descriptor criteria for predicting agonists (Crit-AG); (F) distribution of calculated LELP values for predicting antagonists.
