# Supplementary material for: In Silico Drug Repurposing Framework Predicts Repaglinide, Agomelatine and Protokylol as TRPV1 Modulators with Analgesic Activity
Source: Pharmaceutics. 2022 Nov 22;14(12):2563. doi: 10.3390/pharmaceutics14122563 (PMC9781017; doi:10.3390/pharmaceutics14122563)
Supplement: Supplementary file 1 [file pharmaceutics-14-02563-s001.zip › pharmaceutics-2024941-supplementary.pdf]

pharmaceutics

MDPI

Supplementary Materials: In Silico Drug Repurposing Framework Predicts Repaglinide, Agomelatine and Protokylol as TRPV1 Modulators with Analgesic Activity

Corina Andrei, Dragos Paul Mihai, Anca Zanfirescu, George Mihai Nitulescu and Simona Negres

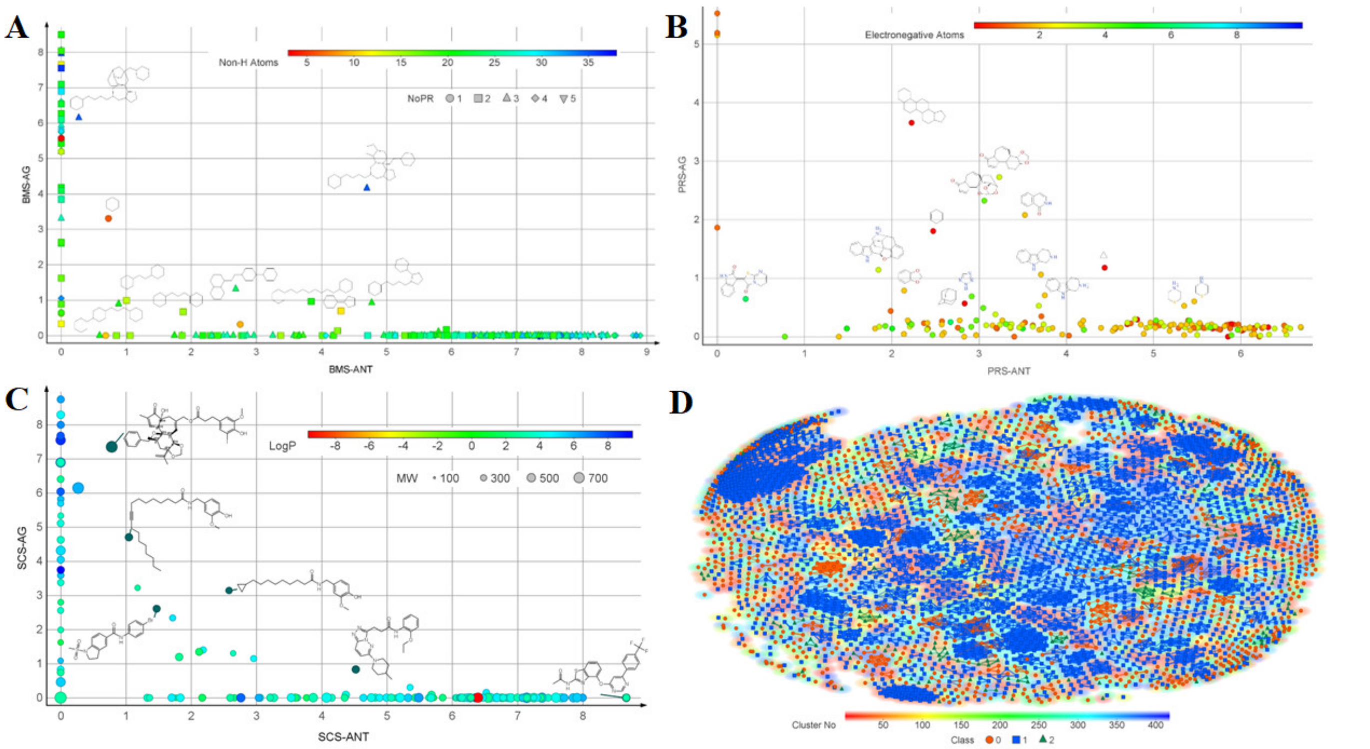

Figure S1. (**A**) Relationship between BM scores for predicting antagonists and agonists; (**B**) relationship between PR scores for predicting antagonists and agonists; (**C**) relationship between SC scores for predicting antagonists and agonists (representative structures are highlighted); (**D**) map of structure similarity relationships based on flexophores for TRPV1 antagonists (class 1), agonists (class 2) and inactive molecules (class 0).

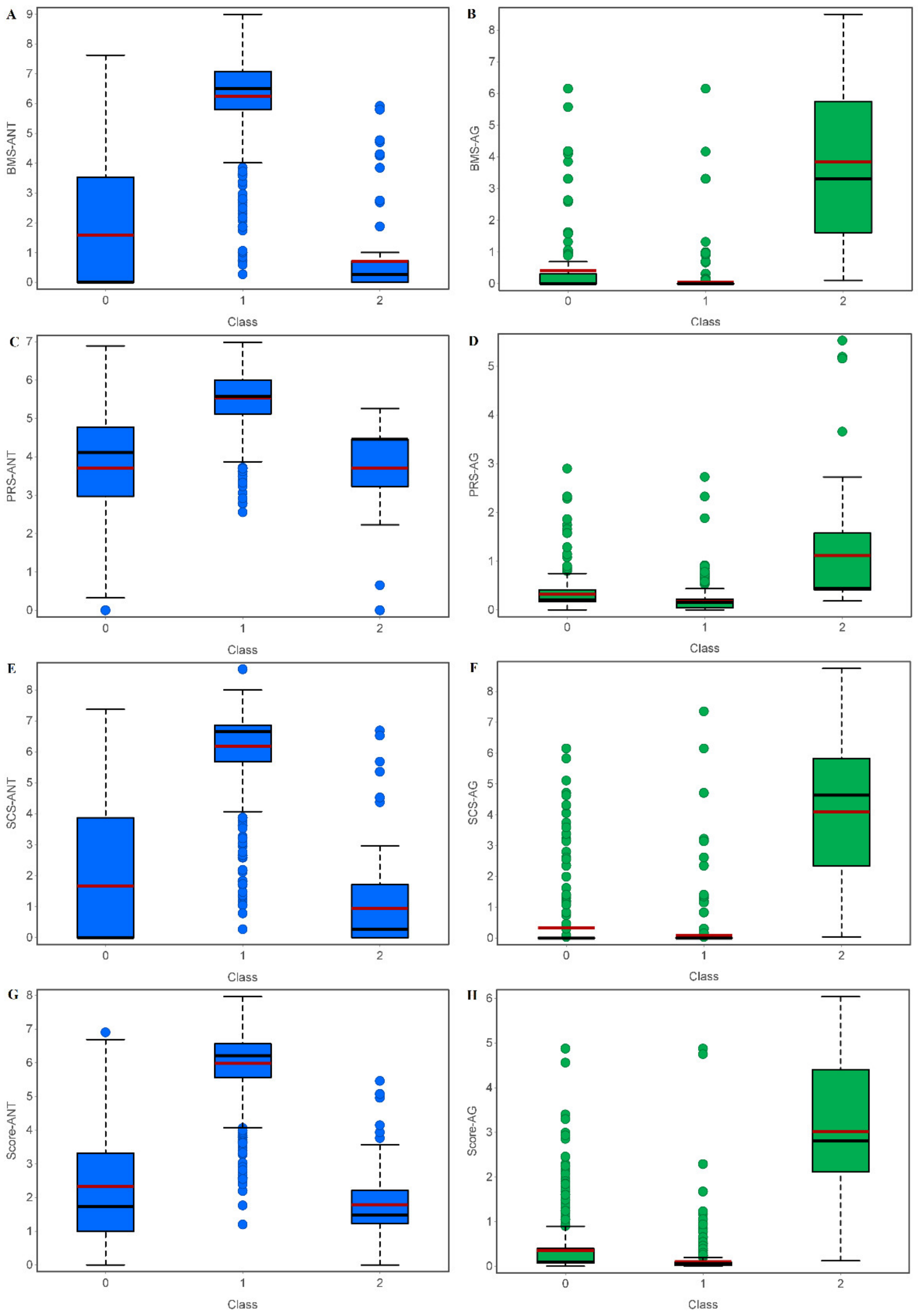

Figure S2. Box plots representing activity scores established based on structural features for both antagonists and agonists. (**A**) Bemis-Murcko activity scores for predicting antagonists (BMS-ANT); (**B**) Bemis-Murcko activity scores for predicting agonists (BMS-AG); (**C**) plain rings activity scores for predicting antagonists (PRS-ANT); (**D**) plain rings activity scores for predicting agonists (PRS-AG); (**E**) flexophore similarity cluster activity scores for predicting antagonists (SCS-ANT); (**F**) flexophore similarity cluster activity scores for predicting agonists (SCS-AG); (**G**) average activity scores for predicting antagonists (Score-ANT); (**H**) average activity scores for predicting agonists (Score-AG); class 0 – inactive molecules, class 1 – antagonists, class 2 – agonists.

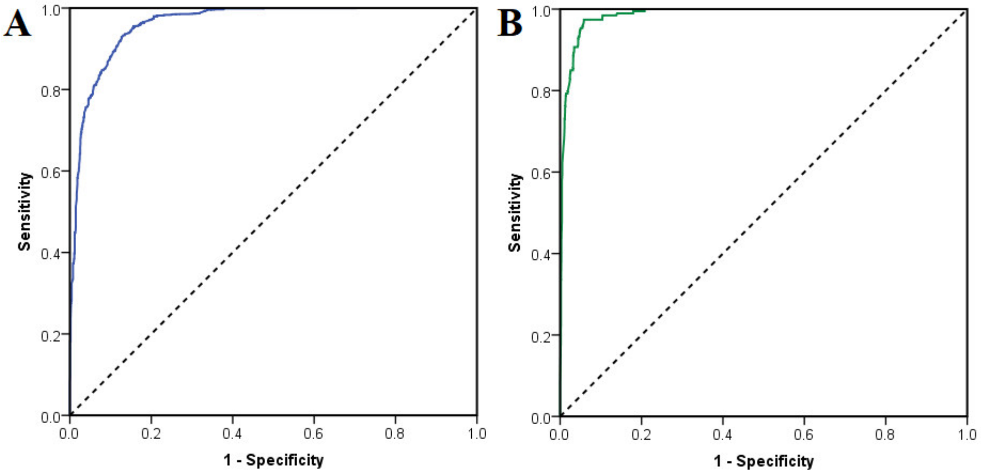

Figure S3. ROC curves showing discriminant capacity of average activity scores. (**A**) activity scores for predicting antagonists (Score-ANT); (**B**) activity scores for predicting agonists (Score-AG).

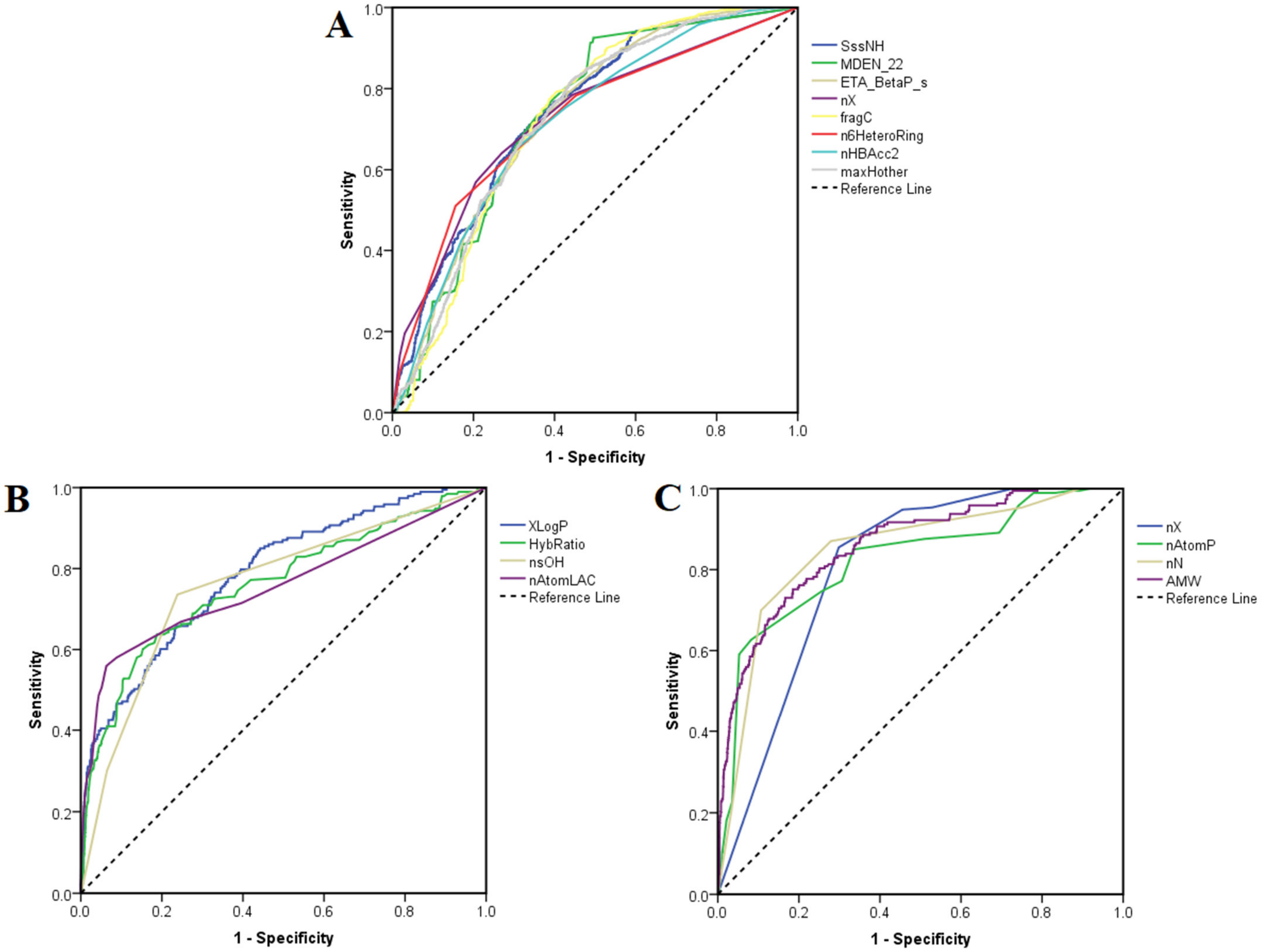

Figure S4. ROC curves showing discriminant capacity of each selected descriptor. (**A**) descriptors included for antagonists classification (larger values indicate more positive test result); (**B**) descriptors included for agonists classification, with higher values indicating more positive test result; (**C**) descriptors included for agonists classification, with lower values indicating more positive test result.

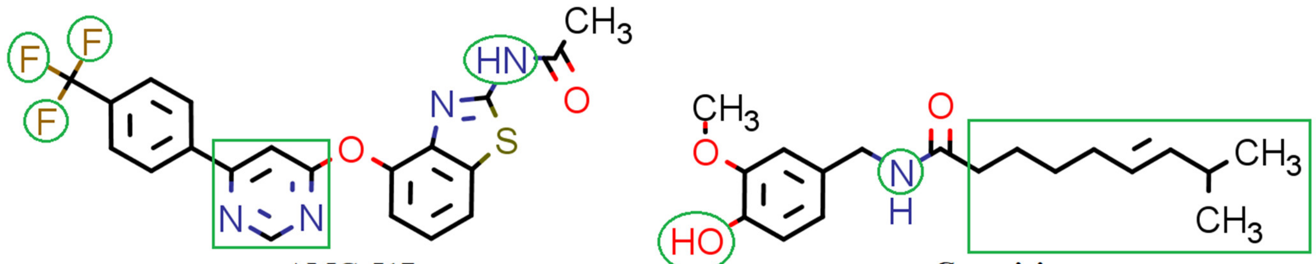

AMG-517 **Capsaicin**

Figure S5. Chemical structures of TRPV1 antagonist AMG-517 and TRPV1 agonist capsaicin.

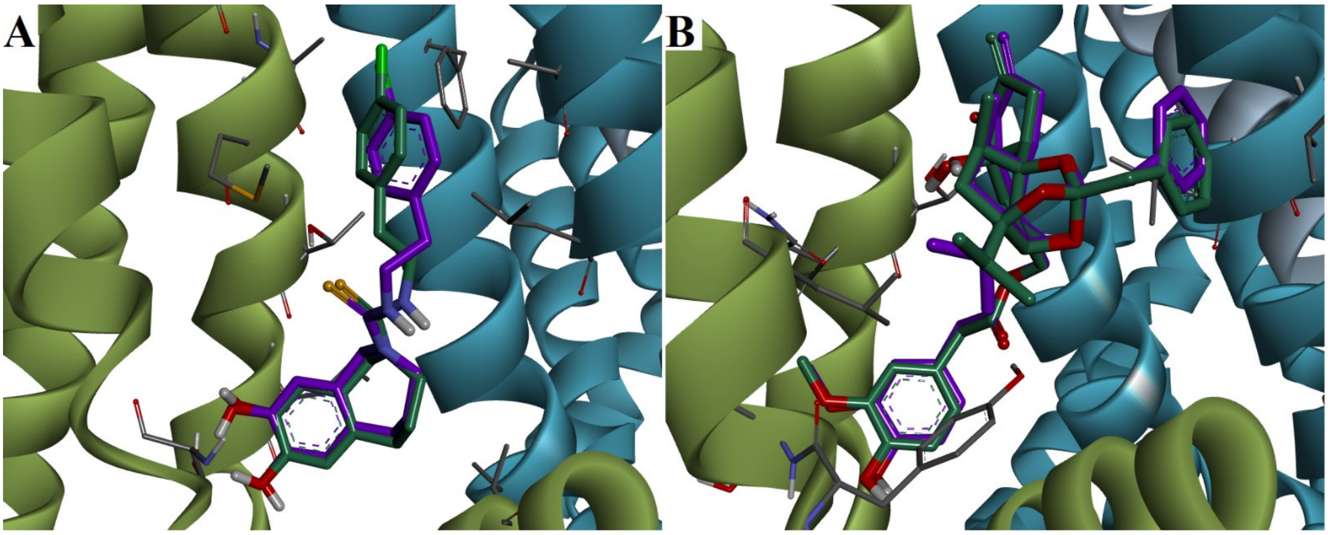

Figure S6. Validation of binding pose prediction. (**A**) superposition between predicted (purple) and experimental (green) conformations for CPZ; (**B**) superposition between predicted (purple) and experimental (green) conformations for RTX.

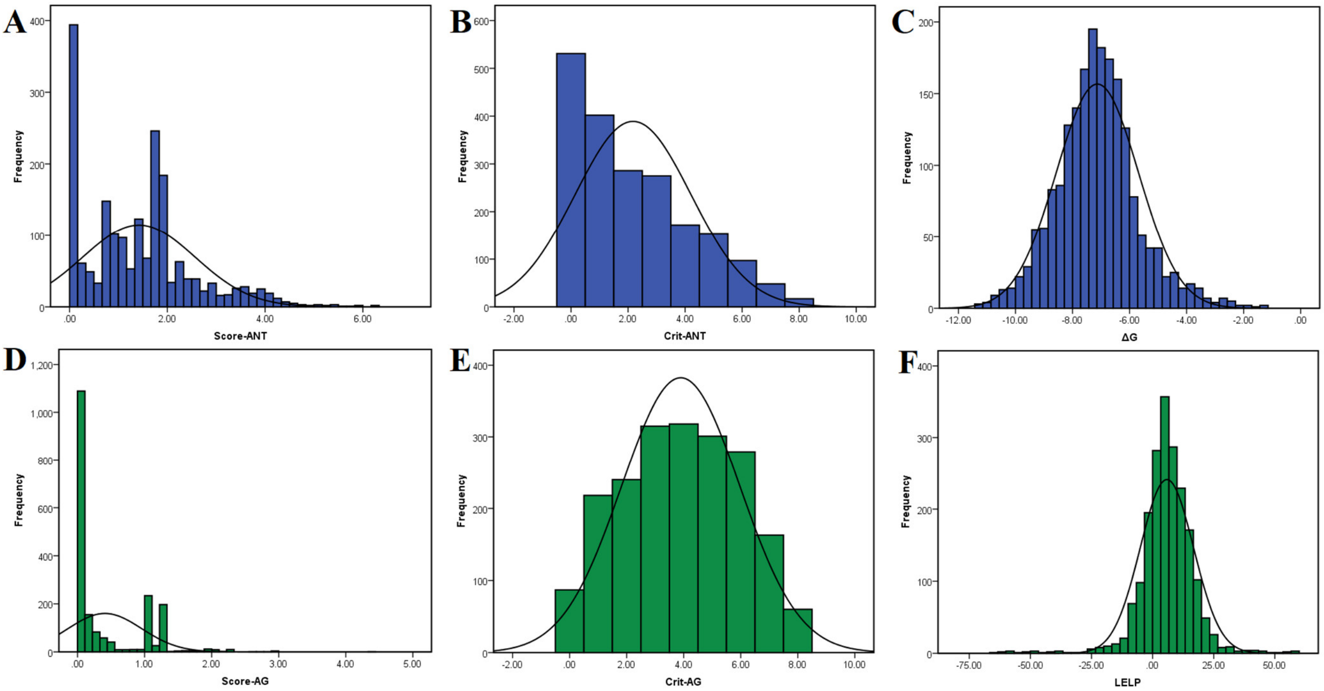

Figure S7. Distribution of input variables established for approved drugs (DrugBank). (**A**) distribution of average activity scores for predicting antagonists (Score-ANT); (**B**) distribution of number of satisfied molecular descriptor criteria for predicting antagonists (Crit-ANT); (**C**) distribution of

binding energies for predicting antagonists (ΔG); (**D**) distribution of average activity scores for predicting agonists (Score-AG); (**E**) distribution of number of satisfied molecular descriptor criteria for predicting agonists (Crit-AG); (**F**) distribution of calculated LELP values for predicting antagonists.
